# Stronger correlation of peak oxygen uptake with distance of incremental shuttle walk test than 6-min walk test in patients with COPD: a systematic review and meta-analysis

**DOI:** 10.1186/s12890-022-01897-0

**Published:** 2022-03-24

**Authors:** Ganghee Chae, Eun Jae Ko, Sei Won Lee, Hyun Jung Kim, Sang Gyu Kwak, Donghwi Park, Seung Won Ra

**Affiliations:** 1 Department of Pulmonary and Critical Care Medicine, Asan Medical Center, University of Ulsan College of Medicine, Seoul, Republic of Korea; 2 Department of Rehabilitation Medicine, Asan Medical Center, University of Ulsan College of Medicine, Seoul, Republic of Korea; 3 Department of Preventive Medicine, College of Medicine, Institute for Evidence-Based Medicine, Korea University, Seoul, Republic of Korea; 4 Department of Medical Statistics, College of Medicine, Catholic University of Daegu, Daegu, Republic of Korea; 5 Department of Physical Medicine and Rehabilitation, Ulsan University Hospital, University of Ulsan College of Medicine, 877 Bangeojinsunhwando-ro, Dong-gu, Ulsan, 44033 Republic of Korea; 6 Division of Pulmonary Medicine, Department of Internal Medicine, Ulsan University Hospital, University of Ulsan College of Medicine, 877 Bangeojinsunhwando-ro, Dong-gu, Ulsan, 44033 Republic of Korea

**Keywords:** Exercise test, Shuttle walk test, Peak oxygen uptake, Chronic obstructive pulmonary disease, Meta-analysis

## Abstract

**Background:**

The 6-min walk test (6MWT) and incremental shuttle walk test (ISWT) are valid and reliable measures to assess exercise capacity of patients with chronic obstructive pulmonary disease (COPD). However, the comparison of correlation between peak oxygen uptake (peak VO_2_) and 6MWT or ISWT distance has not been investigated. We aimed to investigate the correlation between peak VO_2_ and 6MWT and ISWT distances in COPD patients through a meta-analysis.

**Methods:**

We systematically searched MEDLINE, Scopus, Embase, and the Cochrane Library up to June, 2020 for studies comparing the correlation of peak VO_2_ with either 6MWT or ISWT in COPD patients. Meta-analysis was performed with R software using a fixed-effect model. We compared the correlation coefficient and measured the heterogeneity using I^2^ statistics.

**Results:**

We identified 12 studies involving 746 patients. Meta-analysis showed a significant correlation between peak VO_2_ and 6MWT and ISWT distances (6MWT: r = 0.65, 95% CI 0.61–0.70; ISWT: r = 0.81, 95% CI 0.74–0.85; *p* < 0.0001). The heterogeneity was lower in ISWT than in 6MWT (6MWT: I^2^ = 56%, *p* = 0.02; ISWT: I^2^ = 0%, *p* = 0.71). Subgroup analysis showed a higher correlation coefficient in the low exercise capacity group than in the high exercise capacity group in both field tests.

**Conclusions:**

6MWT and ISWT significantly correlated with peak VO_2_. Our findings suggest that ISWT has a stronger correlation with peak VO_2_ than 6MWT. The exercise capacity in COPD patients may affect the strength of the relationship between peak VO_2_ and walking distance in both field tests, suggesting the importance of using various exercise tests.

*Trial registration* CRD 42020200139 at crd.york.ac.uk/prospero/

**Supplementary Information:**

The online version contains supplementary material available at 10.1186/s12890-022-01897-0.

## Background

Chronic obstructive pulmonary disease (COPD) is characterized by airflow limitation that is not fully reversible and often progressive. The airflow limitation is associated with an inflammatory response of the lungs, mainly caused by smoking. Cough, sputum, and dyspnea (upon exertion or at rest) are chronic manifestation of COPD as the disease progresses. Exercise limitation is also a prominent complaint in patients with COPD [1]. Therefore, to assess the functional capacity of patients with chronic airway obstruction, cardiopulmonary exercise testing (CPET) and field tests have been used. CPET provides a global assessment of the integrative exercise response involving the pulmonary, cardiovascular, hematopoietic, neuropsychologic, and skeletal muscle systems; thus, it has been regarded as a gold standard test to assess exercise capacity and response to therapeutic interventions in patients with COPD [2]. However, CPET requires the use of costly exercise equipment, trained technicians, and specialists to interpret the results. Furthermore, some COPD patients with severe dyspnea are unable to undergo tests with equipment measuring expired gas, such as a mouthpiece. Field tests are practical and simple; they do not require complex equipment that can hardly be tolerated or have difficult interpretation. In this regard, field tests, such as the 6-min walk test (6MWT) and the incremental shuttle walk test (ISWT), and CPET are complementary. They play a key role in evaluating functional exercise capacity, assessing prognosis, and evaluating response to treatment [3, 4].

Walking distance during these field tests has been used to indicate the level of disability and cardiopulmonary capacity [5]. Peak oxygen uptake (peak VO_2_) is also regarded as an index of cardiopulmonary capacity, and air analysis during a maximal laboratory exercise test is the most precise method of measuring peak VO_2_ [6]. Previous studies showed a strong correlation between the distance of a field test and peak VO_2_ measured using CPET [7, 8].

6MWT is a practical and simple test that requires a patient to walk at the highest speed tolerated for 6 min. Therefore, 6MWT depends on individually adjusted characteristics, which are determined by patients who are even allowed to stop walking if they wish to [4]. By contrast, ISWT offers a different protocol from the 6MWT, which is incremental and externally paced. It is a 12-level test (1 min at each level) that imposes incremental acceleration through an auditory signal used to control the pace on a 10-m shuttle circuit delineated by two traffic cones. ISWT is also simple, has good reproducibility, and requires no specific ergometers [3, 9]. However, it has not been as widely accepted as 6MWT in clinical practice. We hypothesized that the ISWT would better assess the exercise capacity, and pondered whether the ISWT would better correlate with peak VO_2_ in the CPET than would the 6MWT in COPD patients. To our knowledge, no study has compared the correlation between peak VO_2_ and 6MWT or ISWT distance. Therefore, in this study, we aimed to investigate the correlation between peak VO_2_ and 6MWT and ISWT distances in patients with COPD through a meta-analysis and compared the correlation coefficient for assessing the validity of the 6MWT and ISWT to predict peak VO_2_.

## Methods

### Protocol and registration

This meta-analysis was reported in accordance with the Preferred Reporting Items for Systematic Reviews and Meta-Analyses (PRISMA) statement [10]. The present study was registered in the PROSPERO international prospective register of systematic reviews (CRD 42020200139) and approved by the Institutional Review Board of Ulsan University Hospital (NON2020-001).

### Search strategy and selection criteria

We systematically searched relevant studies published up to June 30, 2020 in MEDLINE, Scopus, Embase, and the Cochrane Library databases. The keywords were chosen by two physicians (GC and DP) and reviewed by keywords from other reviews and articles on similar topics. The following keywords were used to search the database: ‘incremental shuttle walk test’, ‘6 min walk test’, ‘cardiopulmonary exercise testing’, and ‘chronic obstructive pulmonary disease’. Citations of published relevant systematic reviews and meta-analyses were examined to identify further pertinent studies, if any [7, 8]. Two articles published in Japan (written in Japanese) were included [11, 12].

Studies including patients with COPD who underwent CPET and either 6MWT or ISWT were selected. The inclusion criteria were as follows: (1) full-text original article; (2) studies on patients with COPD; (3) studies measuring peak VO_2_ using CPET; and (4) studies investigating the correlation between 6MWT and CPET or between ISWT and CPET. The exclusion criteria were as follows: (1) case reports, case series, and review articles; (2) studies that do not report extractable data available for independent parameters; (3) studies investigating only the correlation between 6MWT and ISWT; and (4) studies on patients with other lung diseases.

### Data extraction

Data from included studies were independently extracted and assessed by two independent reviewers (GC and DP) using a standardized data collection form. Discrepancies were resolved by rechecking the source paper and further discussion among all authors. To investigate the correlations between peak VO_2_ and 6MWT distance or between peak VO_2_ and ISWT distance in patients with COPD, we divided the data extracted according to field test type (6MWT and ISWT).

We extracted the following data from eligible studies: surname of the first author, publication year, number of patients, sex ratio, mean age, the results of 6MWT and ISWT distances (meter), peak VO_2_ (ml min^−1^ kg^−1^ or ml min^−1^), the measurement property of peak VO_2_ in CPET, and the correlation coefficient between peak VO_2_ and distance of field test (6MWT or ISWT).

### Quality assessment

The quality of included studies was assessed using the Methodological Index for Non-Randomized Studies (MINORS) [13]. Each study was assessed using eight criteria (maximum score 16): a clearly stated aim, inclusion of consecutive patients, prospective collection of data, endpoints appropriate to the aim of the study, unbiased assessment of the study endpoint, follow-up period appropriate to the aim of the study, loss to follow up less than 5%, and prospective calculation of the study. The quality of each study was graded as not reported (0), reported but inadequate (1), and reported and adequate (2).

### Statistical analysis

The R Statistics software version 4.0.2 (The R Foundation, Vienna, Austria) was used for statistical analysis of the pooled data. For the meta-analysis, the “meta” and “metafor” packages were used. We analysed the summary of correlation coefficient for validity assessment of 6MWT or ISWT to predict peak VO_2_. In each meta-analysis, I^2^ statistics was used to assess the heterogeneity, providing a measure of extent of inconsistency among results. Statistical significance was set at *p* value < 0.05, and a 95% confidence interval (CI) was used. The risk of publication bias was determined using a funnel plot and the Egger's test. Since the included studies were relatively homogenous patients and estimated that the effect size of the population would be consistent, a fixed-effect model was used. Sensitivity analysis was performed using a random-effects model to explore the effects of computational model. Further, studies that conducted each one of 6MWT, ISWT, and CPET were analysed.

As there was a substantial heterogeneity of correlation coefficiencies between peak VO_2_ and 6MWT distance, subgroup analysis using study-level characteristics was performed [14]. The exercise capacity of populations studied demonstrated a high between-study variability. We, therefore, divided the patients with COPD into low or high exercise capacity group using the cut-off of the peak VO_2_ of 15 ml ∙min^−1^ ∙kg^−1^ or 1000 ml ∙min^−1^ [15, 16].

## Results

### Identification of relevant studies

**Fig. 1:**
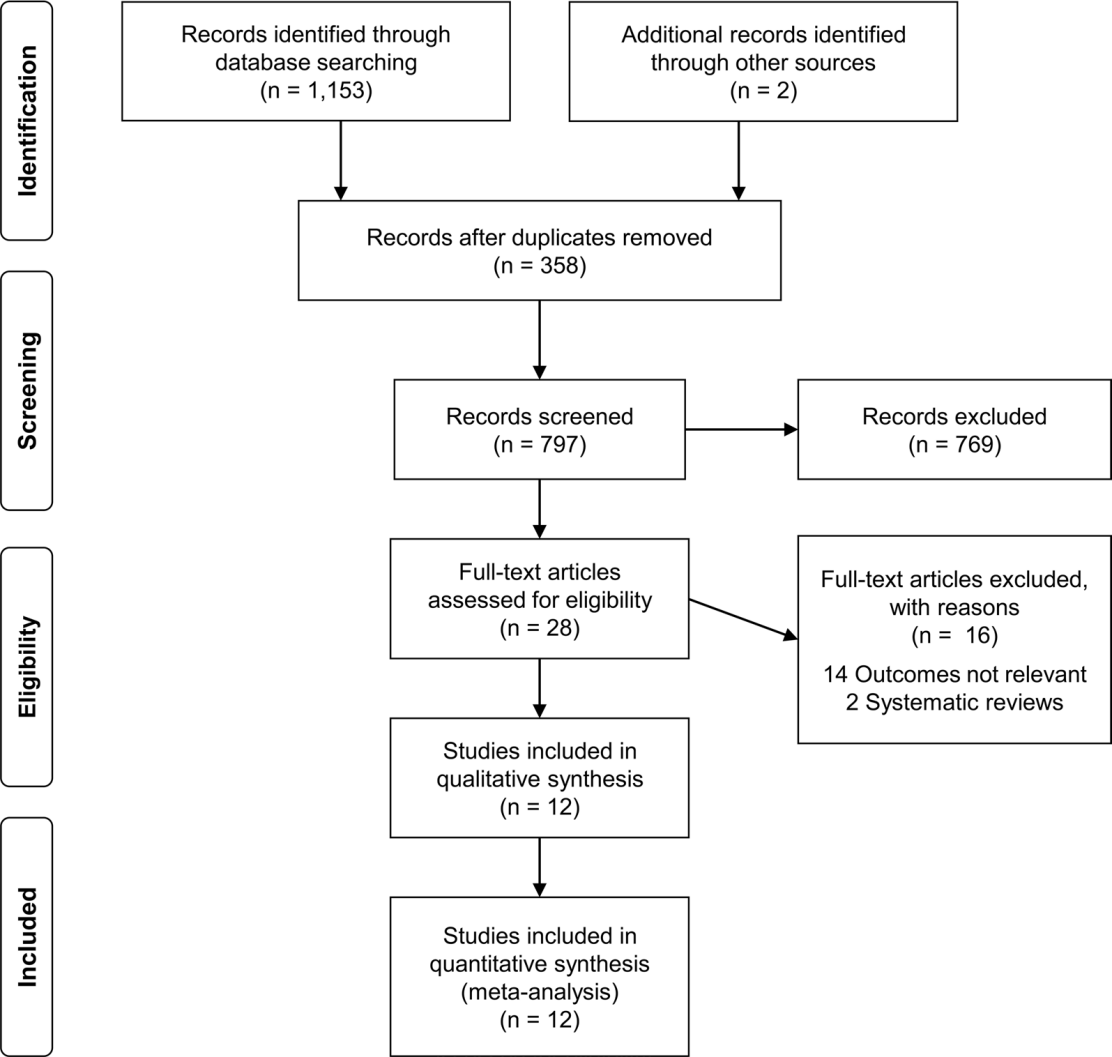
Preferred reporting items for systematic reviews and meta-analyses flow diagram for the identification of studies included in the meta-analysis about the correlation between peak VO_2_ and 6MWT and ISWT distances in patients with COPD. Abbreviations: peakVO_2_, peak oxygen uptake; 6MWT, 6-min walk test; ISWT, incremental shuttle walk test; COPD, chronic obstructive pulmonary disease

A total of 797 published studies were found after removing 358 duplicate articles from 1153 articles searched by keyword, and two additional articles were identified through other sources. Of the 797 retrieved articles, 12 studies were finally included in the meta-analysis after the inclusion and exclusion criteria were applied (Fig. 1) [5, 6, 11, 12, 17–24].

### Study characteristics

Study characteristics and detailed methodologies of included studies are reported in Tables 1 and 2. Twelve studies included a total of 746 patients with COPD, with individual sample size ranging from 13 to 209. According to the type of field test, 10 studies reported correlation between peak VO_2_ and 6MWT distance and five studies reported correlation between peak VO_2_ and ISWT distance.

**Table 1:** Characteristics of the included studies

Study [ref.]	Year	No. of patients	Sex (M/F)	Mean age	6MWT distance (m)	Peak VO_2_		CPET measurement	Correlation coefficient
						(ml min^−1^ kg^−1^)	(ml min^−1^)		
Rejeski [17]	2000	209	117/92	67.2 ± 6.0	474.9 ± 105.2	17.13 ± 4.1		Treadmill	0.64^§^
Chuang [18]	2001	27	27/0	65 ± 6	456.0 ± 84.0	18.9 ± 5.0	1110.0 ± 350.0	Cycle ergometer	0.37^§^
Arizono [11]	2002	17	15/2	71.1 ± 4.3	427.5 ± 61.9	15.2 ± 2.8		Treadmill	0.577^§^
Oga [19]	2002	36	36/0	69 ± 7	492.0 ± 66.0	15.6 ± 3.7		Cycle ergometer	0.64^§^
Carter [20]	2003	124	90/34	66.8 ± 7.3	403.0 ± 81.6		1095.6 ± 323.5	Cycle ergometer	0.54^†^
Turner [5]	2004	20	15/5	64.0 ± 7.5	474.9 ± 87.8	14.2 ± 2.9		Cycle ergometer	0.73^§^
Starobin [21]	2006	50	28/22	64.3 ± 11.7	434.7 ± 88.0	13.5 ± 4.1		Cycle ergometer	0.58^§^
Arizono [12]	2008	100	91/9	69.6 ± 9.7	479.7 ± 122.5	12.6 ± 3.2		Cycle ergometer	0.773^§^
Hill [22]	2008	50	36/14	68 ± 8	464.0 ± 110.0		874.0 ± 243.0	Cycle ergometer	0.63^§^
Díaz [23]	2010	81	49/32	67 ± 8	514.0 ± 85.0	16.5 ± 4.0		Cycle ergometer	0.78^§^

Reporting correlation between peak VO_2_ and 6MWT distance in patients with COPD (n = 10)

Data are presented as mean ± SD, unless otherwise stated

6MWT, 6-Minute walk test; peak VO_2_, peak oxygen uptake; COPD, chronic obstructive pulmonary disease; CPET, cardiopulmonary exercise test

^§^Pearson’s r;

^†^Spearman's rho

**Table 2:** Characteristics of the included studies reporting correlation between peak VO_2_ and ISWT distance in patients with COPD (n = 5)

Study [ref.]	Year	No. of patients	Sex (M/F)	Mean age	ISWT distance (m)	Peak VO_2_ (ml min^−1^ kg^−1^)	CPET measurement	Correlation coefficient
Singh [6]	1994	19	17/2	61 ± 7	401.8 ± 142.3	14.2 ± 4.1	Treadmill	0.88^§^
Arizono [11]	2002	17	15/2	71.1 ± 4.3	353.5 ± 123.3	15.2 ± 2.8	Treadmill	0.776^§^
Onorati [24]	2003	13	13/0	70 ± 1	391.0 ± 31.0	19.1 ± 0.8	Cycle ergometer	0.72^§^
Turner [5]	2004	20	15/5	64.0 ± 7.5	339.0 ± 97.2	14.2 ± 2.9	Cycle ergometer	0.73^§^
Arizono [12]	2008	100	91/9	69.6 ± 9.7	378.2 ± 152.2	12.6 ± 3.2	Cycle ergometer	0.813^§^

Data are presented as mean ± SD, unless otherwise stated

ISWT, incremental shuttle walk test; peak VO_2_, peak oxygen uptake; COPD, chronic obstructive pulmonary disease; CPET, cardiopulmonary exercise test

^§^Pearson's r

### Quality assessment

In all included studies, ‘a clearly stated aim’, ‘inclusion of consecutive patients’, ‘endpoints appropriate to the aim of the study’, ‘unbiased assessment of the study endpoint’, and ‘follow-up period appropriate to the aim of the study’ were reported adequately. However, regarding ‘prospective collection of data’, only one study reported inappropriately. More than 5% loss to follow-up was reported in three studies. Any included studies did not calculate the appropriate sample size prior to the initiation of each study (Additional file 1: Table S1).

### Meta-analysis results

**Fig. 2:**
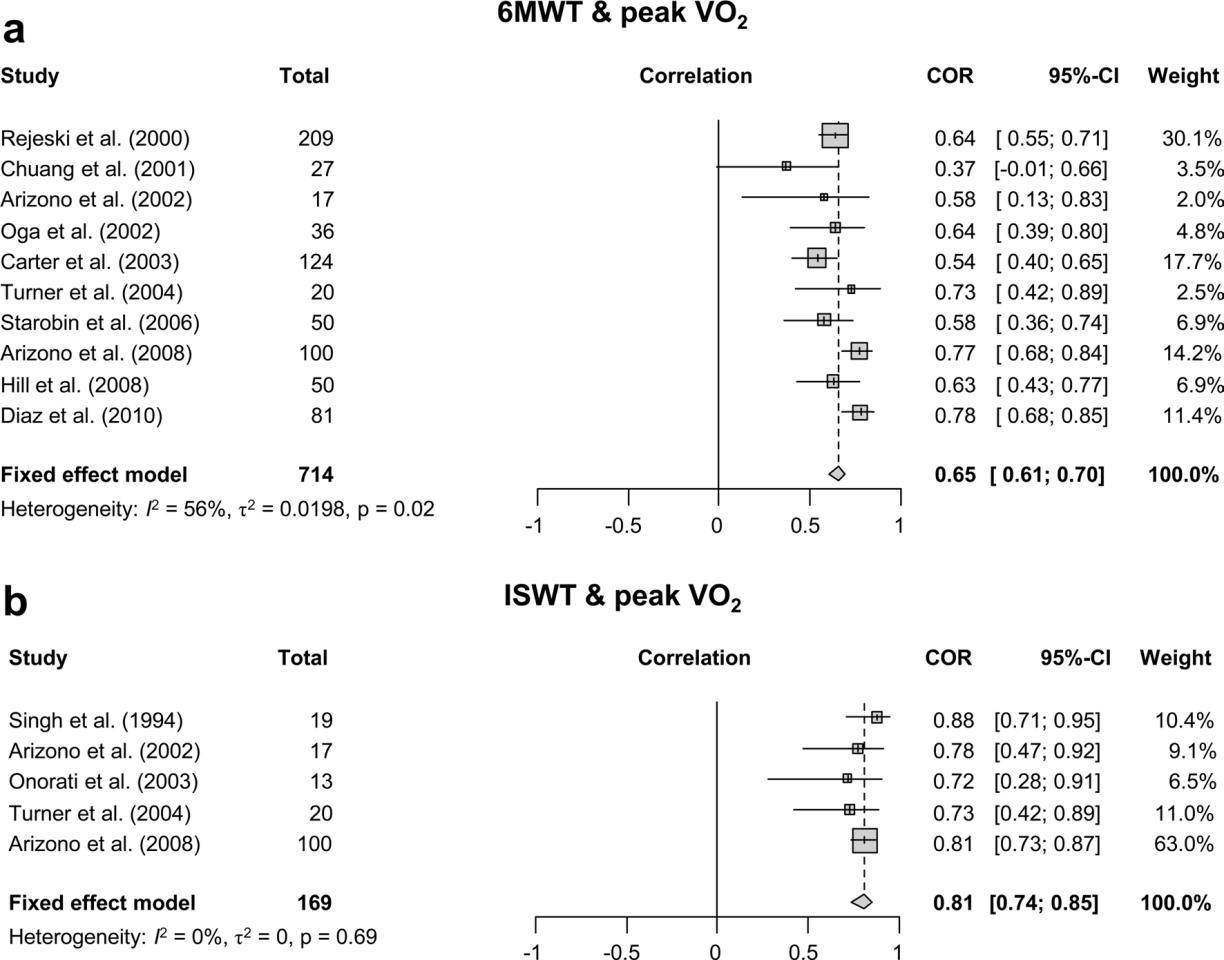
Forest plot of meta-analysis. Meta-analysis was performed in **A** 10 studies assessing the correlation coefficient between peak VO_2_ and distance of the 6MWT and in **B** five studies assessing the correlation coefficient between peak VO_2_ and ISWT distance. 6MWT, 6-min walk test; peak VO_2_, peak oxygen uptake; ISWT, incremental shuttle walk test

In patients with COPD who underwent CPET and 6MWT (10 studies, n = 714), the 6MWT distance was a significant predictor of peak VO_2_ measured using CPET (r = 0.65, 95% CI 0.61 to 0.70). In patients with COPD who underwent CPET and ISWT (5 studies, n = 169), the ISWT distance was also a significant predictor of peak VO_2_ measured using CPET (r = 0.81, 95% CI 0.74 to 0.85). The heterogeneity was higher in 6MWT than in ISWT (6MWT: I^2^ = 56%; *p* = 0.02, ISWT: I^2^ = 0%; *p* = 0.69) (Fig. 2).

Sensitivity analysis was conducted to evaluate the effects of computational model. The heterogeneity, pooled correlation coefficients, and 95% CI of the indexes remained stable and were not significantly altered by the random-effects model (6MWT: r = 0.65, 95% CI 0.57–to 0.72, ISWT: r = 0.81, 95% CI 0.74– to 0.85; *p* < 0.0001) (Additional file 2: Fig. S1. We performed sensitivity analysis on three studies that conducted each one of 6MWT, ISWT, and CPET. The ISWT also showed a stronger correlation than 6MWT (6MWT: r = 0.75; 95% CI 0.66– to 0.82, ISWT: r = 0.80; 95% CI 0.73–to 0.85, *p* < 0.0001) (Additional file 3: Fig. S2).

**Fig. 3:**
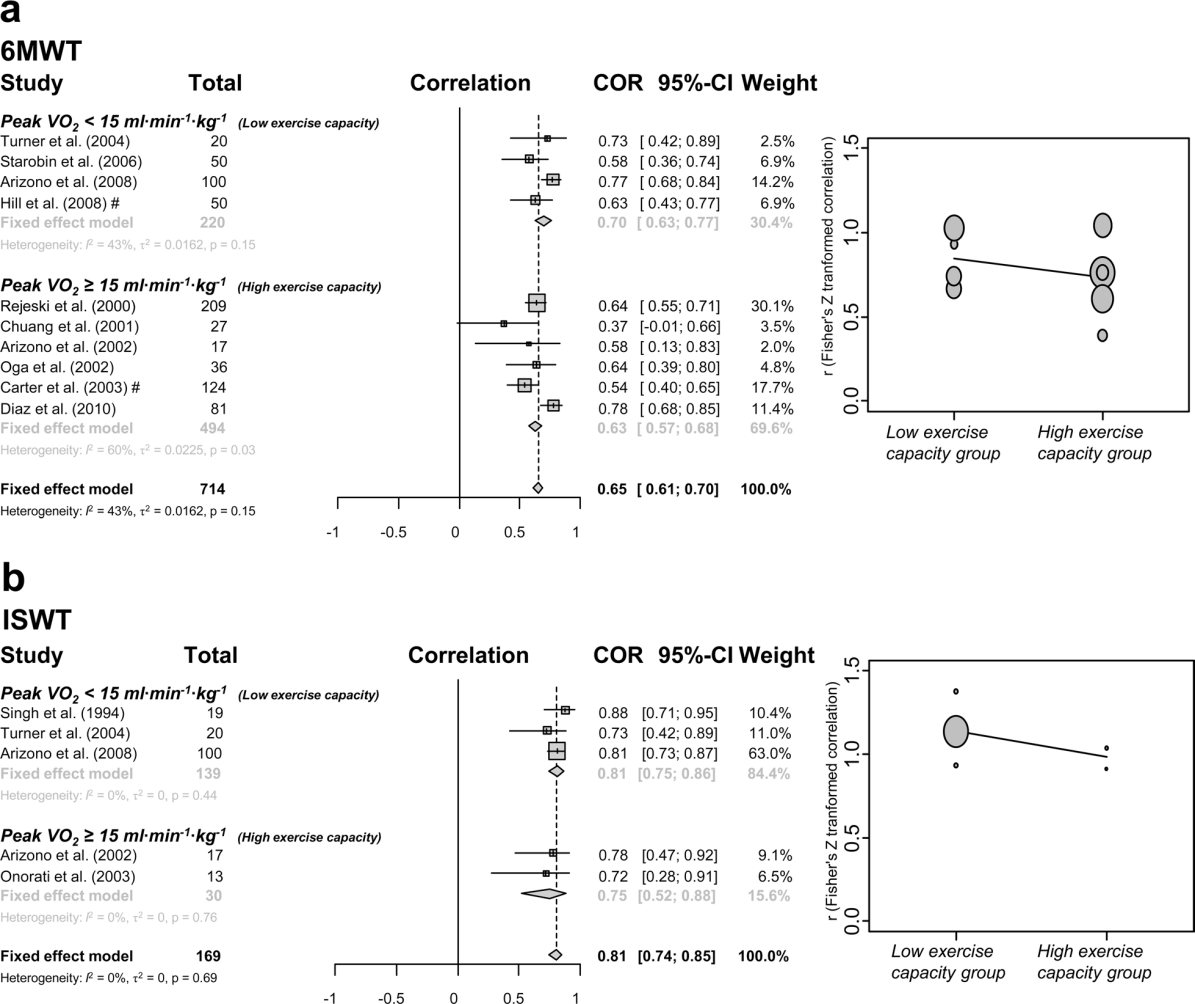
Forest plot and bubble plot of subgroup analysis divided by exercise capacity. #: In this study, we used the cut-off of the peak VO_2_ for 1000 ml∙min^−1^. Abbreviations: 6MWT, 6-min walk test; peak VO_2_, peak oxygen uptake; ISWT, incremental shuttle walk test

We performed a subgroup analysis for exercise capacity of patients with COPD according to the mean of peak VO_2_: low exercise capacity group, peak VO_2_ < 15 ml ∙min^−1^ kg^−1^ or 1000 ml min^−1^; and high exercise capacity group, peak VO_2_ ≥ 15 ml min^−1^ kg^−1^ or 1000 ml min^−1^. In both field tests, the correlation coefficient was higher in the low exercise capacity group than in the high exercise capacity group (low exercise capacity group: r = 0.70 [6MWT], r = 0.81 [ISWT]; *p* = 0.017, high exercise capacity group: r = 0.63 [6MWT], r = 0.75 [ISWT]; *p* = 0.256). The heterogeneity of 6MWT in the high exercise capacity group was higher than in the low exercise capacity group (low exercise capacity group: I^2^ = 43%; *p* = 0.15, high exercise capacity group: I^2^ = 60%; *p* = 0.03). The heterogeneity of ISWT was very low and consistent regardless of peak VO_2_ (low exercise capacity group: I^2^ = 0%; *p* = 0.44, high exercise capacity group: I^2^ = 0%; *p* = 0.76) (Fig. 3).

### Publication bias

**Fig. 4:**
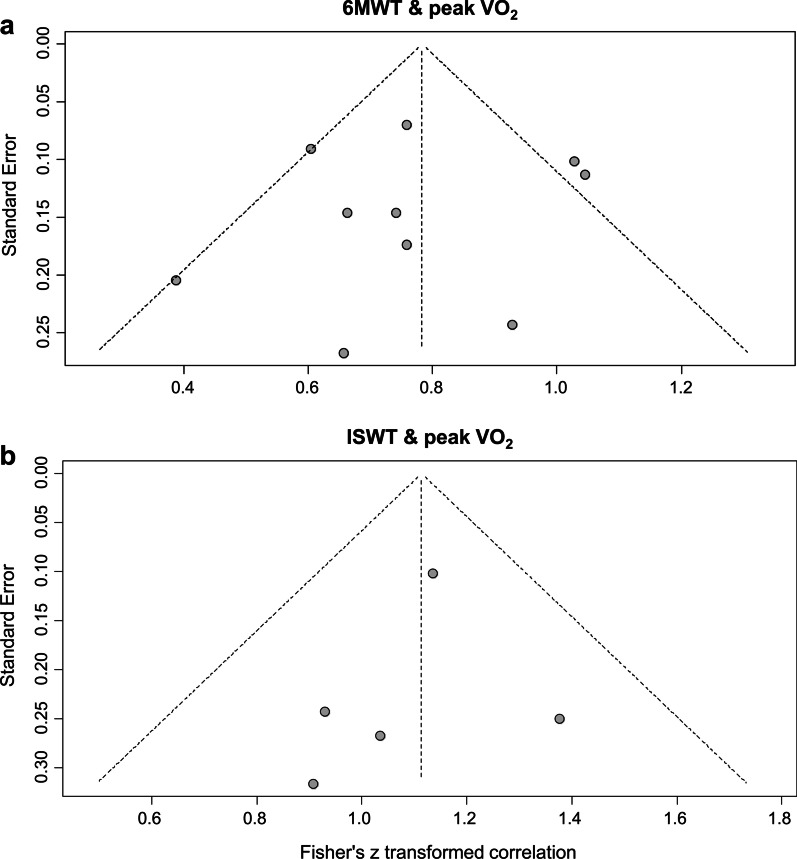
Linear regression test of funnel plot asymmetry for identifying publication bias. Abbreviations: 6MWT, 6-min walk test; peak VO_2_, peak oxygen uptake; ISWT, incremental shuttle walk test

A funnel plot was created to investigate the risk of publication bias. In patients with COPD who underwent CPET and 6MWT, a funnel plot between peak VO_2_ and 6MWT distance showed some asymmetry. However, the Egger's test showed no statistical significance (*p* = 0.73). These findings indicate that the asymmetry observations were not supported and that there may be no risk of publication bias. In patients with COPD who underwent CPET and ISWT, a funnel plot between peak VO_2_ and ISWT distance showed symmetry. The Egger's test showed no statistical significance (*p* = 0.62), indicating that there may be no risk of publication bias (Fig. 4).

## Discussion

Several studies have compared the correlation between peak VO_2_ and ISWT and 6MWT distances, but this study is the first to perform a meta-analysis of the correlation. This meta-analysis provided further evidence that the 6MWT and ISWT distances had significant correlations with peak VO_2_ measured using CPET in patients with COPD. When comparing the correlation coefficients with peak VO_2_, the ISWT showed a stronger correlation than the 6MWT. These results suggest that ISWT reflects a stronger correlation with peak VO_2_ measured using CPET than 6MWT in patients with COPD.

To date, many studies have confirmed that 6MWT and ISWT are valid, reliable, and responsive to therapeutic interventions [8, 25, 26]. However, the 6MWT and ISWT entail substantially different protocols. The 6MWT is a self-paced submaximal test and can be performed continuously or intermittently. This test is sensitive to methodological variations, such as encouragement, oxygen supplement, and circumstances (e.g., wheeled walking aid, circular/straight track, indoors/outdoors) [26]. By contrast, ISWT is an externally paced maximal exercise test [5, 8, 26]. This feature may be an advantage in circumstances where methodological variation is a concern if the test is performed by various sites or operators [8]. In addition, where the larger space requirements of 6MWT preclude its use, ISWT can be a useful alternative [8]. 6MWT requires a 30-m walking course, but ISWT only requires a short course of 10-m walking course. The protocol of ISWT is more standardized than that of 6MWT, and the proposal of incremental values is also clear in the shuttle walk test. In addition, ISWT shows a linear change of lung gas exchange including peak VO_2_, but 6MWT shows an exponential change [24]. Furthermore, the walking distance in ISWT has been reported to be reliable and a good indicator for predicting re-hospitalisations in patients with moderate to severe COPD [27]. Therefore, ISWT has better features than 6MWT.

Thus, we aimed to determine the correlation between peak VO_2_ and 6MWT and ISWT distances through a meta-analysis and to compare the correlation coefficient of both field tests. In this study, both field tests were confirmed to show a relatively strong correlation with peak VO_2_ through a meta-analysis. Notably, the correlation coefficient of ISWT was stronger than that of 6MWT. In a subgroup analysis, we found that both field tests had lower correlation coefficients in the high exercise capacity group. That is, when the exercise capacity was good, the correlation between the distance of field tests and peak VO_2_ decreased. This may be related to the “ceiling effect” that occurred in 6MWT. In a related study comparing bronchodilator-induced changes in exercise capacity with the 6MWT and the shuttle walk test, 6MWT showed less responsive for detecting changes in COPD patients with high exercise capacity [28]. In addition, the heterogeneity of ISWT was very low in all analyses. Although there was a limitation to the small number of the included ISWT studies, the low heterogeneity could support the good reproducibility of ISWT. On the contrary, 6MWT showed high heterogeneity, particularly in the high exercise capacity group. ISWT was proven superior to 6MWT for evaluating COPD patients with high exercise capacity.

The 6MWT is reported as a valid and reliable field test and reflects performance of more activities of daily life [4]. A previous study found that changes in dyspnea grade, patient-reported outcomes, 6-min walk distance (6MWD), and cycle endurance time (constant work-rate test), after pulmonary rehabilitation, did not show synchronous responses in patients with COPD, suggesting the need for various tests to evaluate the response in exercise intolerance [29]. Therefore, we proposed that 6MWT, ISWT, and CPET be used complementary to each other to evaluate the exercise capacity of patients with COPD. In our results, the correlation with peak VO_2_ and the heterogeneity of the ISWT were higher, and the heterogeneity of the 6MWT was higher especially in the high exercise capacity group. Therefore, we cautiously recommend performing the ISWT in patients with high exercise capacity and the 6MWT in those with low exercise capacity. The most appropriate exercise tests should be applied, depending on each COPD patient’s exercise capacity to evaluate their underlying pathophysiology of exercise intolerance.

Despite the advantages of the ISWT, it is underutilized to evaluate exercise capacity and effectiveness of treatment and respiratory rehabilitation and to predict prognosis in chronic respiratory diseases. Many studies have already demonstrated its superiority in preoperative evaluation, respiratory rehabilitation, and cardiopulmonary function evaluation. However, the ISWT has not been introduced in many countries, including South Korea. Thus, adoption of various complementary tests and evaluation of exercise capacity with an appropriate test will enable comprehensive assessment and provision of an individualized exercise program in patients with COPD nature.

This systematic review and meta-analysis have several limitations that should be mentioned. Firstly, we only included a small number of studies. Secondly, in the included studies, exercise modalities in CPET were heterogeneous. Peak VO_2_ was measured using a treadmill in three studies and a cycle ergometer in nine studies. Several studies have reported that the peak VO_2_ measured using a cycle ergometer is lower than that using a treadmill, but both peak VO_2_ measured with these devices show a significant correlation [30, 31]. In addition, the objective of this study was not to analyse the absolute value of the peak VO_2_, but to investigate the correlation between the peak VO_2_ and the field test results. Therefore, the difference in CPET exercise method is believed to not significantly affect the results of this study. Finally, for more convincing evidence with regard to the correlation between peak VO_2_ and field tests, more qualified prospective studies are required. In addition, future studies should verify whether the correlation coefficient of ISWT is superior to that of 6MWT as a primary outcome after calculating the sample size with the superiority study design.

## Conclusions

The present study revealed further evidence that 6MWT and ISWT correlated significantly with peak VO_2_ measured using CPET in patients with COPD through a meta-analysis. Moreover, the ISWT distance showed a stronger correlation with peak VO_2_ than did the 6MWT distance as expected, suggesting that ISWT is more valid and reliable for assessing the maximal exercise capacity in patients with COPD. However, the strength of the relationship between peak VO_2_ and walking distance in the field tests was affected by the exercise capacity in COPD patients, suggesting the importance of using various exercise tests.

## Supplementary Information

**Additional file 1. Table S1: **Results of quality assessment for included studies using the Methodological Index for Non-Randomized Studies.

**Additional file 2. Figure S1: **Forest plot of sensitivity analysis performed using a random-effects model. 6MWT, 6-minute walk test; peak VO2, peak oxygen uptake; ISWT, incremental shuttle walk test.

**Additional file 3. Figure S2: **Forest plot of sensitivity analysis performed on three studies that conducted each one of 6MWT, ISWT, and CPET. 6MWT, 6-minute walk test; ISWT, incremental shuttle walk test; CPET, cardiopulmonary exercise testing; peak VO2, peak oxygen uptake.

## Data Availability

The datasets used and/or analysed during the current study are available from the corresponding author on reasonable request.
