# Supplementary material for: Stronger correlation of peak oxygen uptake with distance of incremental shuttle walk test than 6-min walk test in patients with COPD: a systematic review and meta-analysis
Source: BMC Pulm Med. 2022 Mar 24;22:102. doi: 10.1186/s12890-022-01897-0 (PMC8953060; doi:10.1186/s12890-022-01897-0)
Supplement: Supplementary file 1 — Additional file 1. Table S1: Results of quality assessment for included studies using the Methodological Index for Non-Randomized Studies. [file 12890_2022_1897_MOESM1_ESM.doc]

**Table S1** Results of quality assessment for included studies using the Methodological Index for Non-Randomised Studies (MINORS)

| **Study [ref.]** | **A clearly stated aim** | **Inclusion of consecutive patients** | **Prospective collection of data** | **Endpoints appropriate to the aim of the study** | **Unbiased assessment of the study endpoint** | **Follow-up period appropriate to the aim of the study** | **Loss to follow up less than 5%** | **Prospective calculation of the study size** | **Total** |
| --- | --- | --- | --- | --- | --- | --- | --- | --- | --- |
| Singh [6] | 2 | 2 | 2 | 2 | 2 | 2 | 2 | 0 | 14 |
| Rejeski [17] | 2 | 2 | 1 | 2 | 2 | 2 | 2 | 0 | 13 |
| Chuang [18] | 2 | 2 | 2 | 2 | 2 | 2 | 1 | 0 | 13 |
| Arizono [11] | 2 | 2 | 2 | 2 | 2 | 2 | 2 | 0 | 14 |
| Oga [19] | 2 | 2 | 2 | 2 | 2 | 2 | 1 | 0 | 13 |
| Carter [20] | 2 | 2 | 2 | 2 | 2 | 2 | 2 | 0 | 14 |
| Onorati [24] | 2 | 2 | 2 | 2 | 2 | 2 | 2 | 0 | 14 |
| Turner [5] | 2 | 2 | 2 | 2 | 2 | 2 | 2 | 0 | 14 |
| Starobin [21] | 2 | 2 | 2 | 2 | 2 | 2 | 2 | 0 | 14 |
| Arizono [12] | 2 | 2 | 2 | 2 | 2 | 2 | 2 | 0 | 14 |
| Hill [22] | 2 | 2 | 2 | 2 | 2 | 2 | 1 | 0 | 13 |
| Díaz [23] | 2 | 2 | 2 | 2 | 2 | 2 | 2 | 0 | 14 |

The items are scored 0 (not reported), 1 (reported but inadequate), or 2 (reported and adequate).
