# Supplementary figures and images for: Stronger correlation of peak oxygen uptake with distance of incremental shuttle walk test than 6-min walk test in patients with COPD: a systematic review and meta-analysis
Source: BMC Pulm Med. 2022 Mar 24;22:102. doi: 10.1186/s12890-022-01897-0 (PMC8953060; doi:10.1186/s12890-022-01897-0)

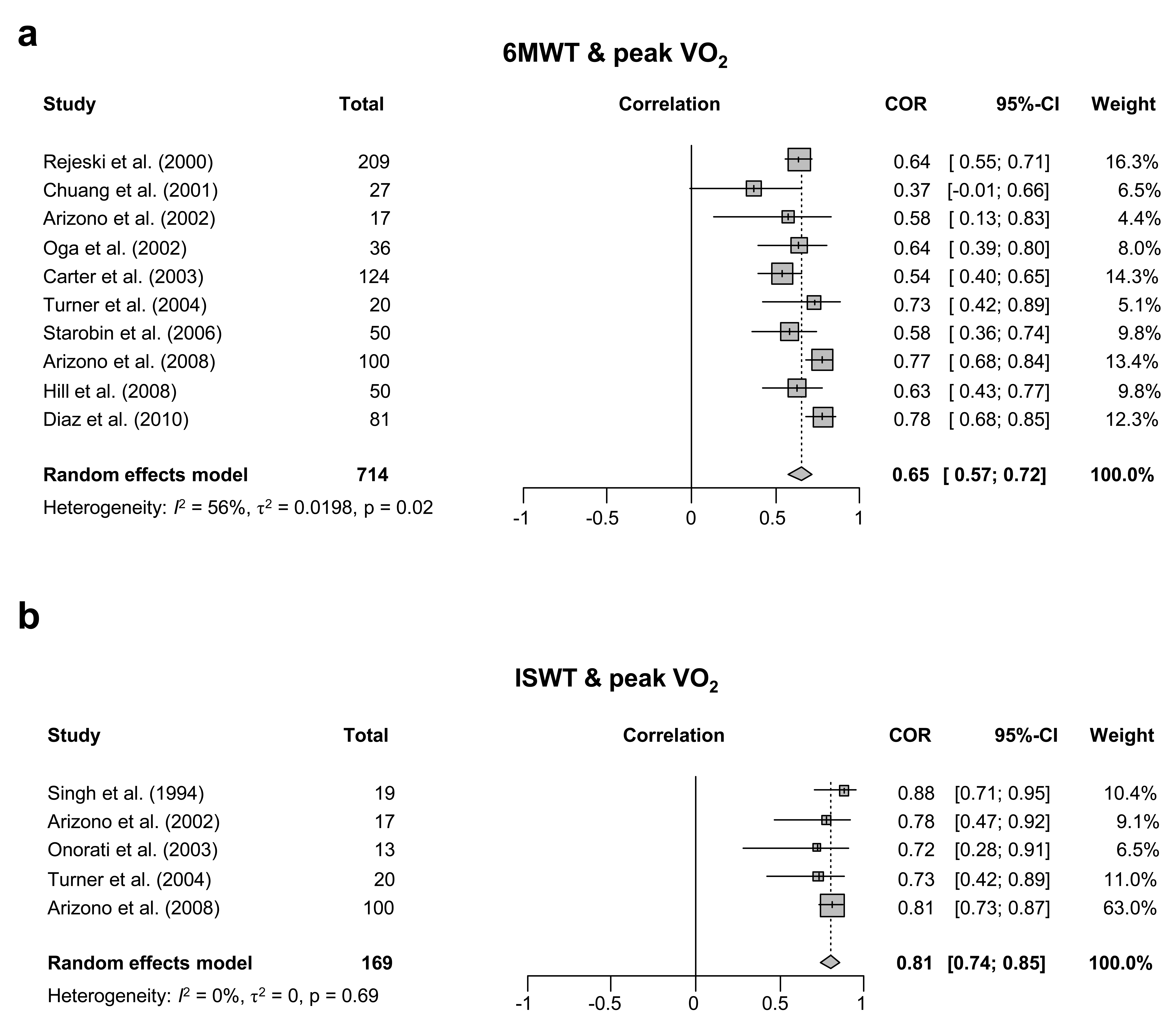

Supplement: Supplementary file 2 — Additional file 2. Figure S1: Forest plot of sensitivity analysis performed using a random-effects model. 6MWT, 6-minute walk test; peak VO2, peak oxygen uptake; ISWT, incremental shuttle walk test. [file 12890_2022_1897_MOESM2_ESM.tif]

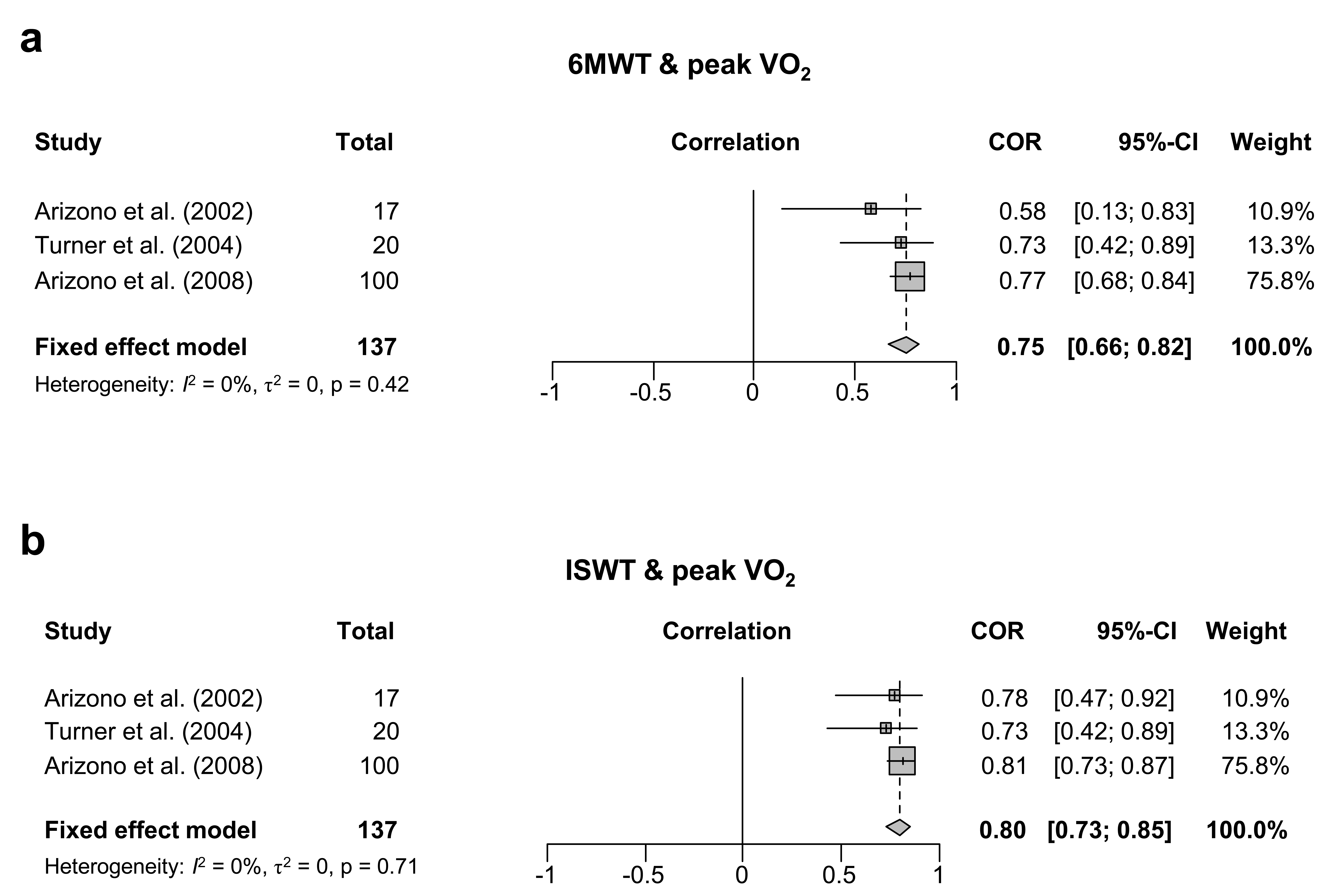

Supplement: Supplementary file 3 — Additional file 3. Figure S2: Forest plot of sensitivity analysis performed on three studies that conducted each one of 6MWT, ISWT, and CPET. 6MWT, 6-minute walk test; ISWT, incremental shuttle walk test; CPET, cardiopulmonary exercise testing; peak VO2, peak oxygen uptake. [file 12890_2022_1897_MOESM3_ESM.tif]
